# Anxiety from a Phylogenetic Perspective: Is there a Qualitative Difference between Human and Animal Anxiety?

**DOI:** 10.1155/2007/59676

**Published:** 2007-06-12

**Authors:** Catherine Belzung, Pierre Philippot

**Affiliations:** ^1^EA3248 Psychobiologie des Émotions, UFR Sciences et Techniques, Université François-Rabelais, Tours 37200, France; ^2^Department of Psychology, Université Catholique de Louvain, 10 place Mercier, 1348 Louvain-la-Neuve, Belgium

## Abstract

A phylogenetic approach to anxiety is proposed. The different facets of human anxiety and their presence at different levels of the phylum are examined. All organisms, including unicellular such as protozoan, can display a specific reaction to danger. The mechanisms enabling the appraisal of harmful stimuli are fully present in insects. In higher invertebrates, fear is associated with a specific physiological response. In mammals, anxiety is accompanied by specific cognitive responses. The expression of emotions diversifies in higher vertebrates, only primates displaying facial expressions. Finally, autonoetic consciousness, a feature essential for human anxiety, appears only in great apes. This evolutive feature parallels the progress in the complexity of the logistic systems supporting it (e.g., the vegetative and central nervous systems). The ability to assess one's coping potential, the diversification of the anxiety responses, and autonoetic consciousness seem relevant markers in a phylogenetic perspective.

## 1. INTRODUCTION

In human, anxiety is present in most psychopathological conditions [1]. The regulation and alleviation of anxiety is a key factor in the promotion of human well-being. However, anxiety is often experienced as an automatic and uncontrollable response with deep roots in our phylogenetic past. On the other hand, psychological processes like rumination that are central to human anxiety, imply high-order cognitive capacities, such as self-consciousness. It thus appears that anxiety comprises many facets, some of which having deep roots in our evolutionary history and others being properly human. From this perspective, a phylogenetic approach to anxiety might deepen our understanding of this phenomenon in human, and help to distinguish similarities and differences with alike states in animals. Further, as progress in the understanding of the neurobiological substrates of anxiety and in the discovery of new pharmacological treatments of anxiety often involves rodent models [2, 3], it is essential to be aware of the processes that are absent in the animal species used [4], in order to be aware of the limits of such models.

To achieve this goal, we used a comparative approach, which consisted in assessing in animal species the presence of the process described in psychology and thus designed for humans. Such rationale provides operational criteria for the study of emotions in animals and may be a heuristic framework for interspecies comparison, which may be used also for emotions other than fear and anxiety. This approach is necessarily theoretical and requires to review many findings obtained in animals research, trying to analyze data obtained within other frames. To this aim, the present paper first describes the different elements constituting human anxiety and examines their presence along the phylum. Then, it reviews the different neurological and physiological systems of the organism supporting the anxiety responses along the phylum. Finally, the different conjunctions in a given species of the elements constituting anxiety will be examined.

## 2. THE DEFINITION OF ANXIETY AND RELATED CONCEPTS

Fear, anxiety, and panic are three related concepts that need to be differentiated. Fear is considered by most emotion theorists as a basic emotion in humans (e.g., [5, 6]). As such, fear would develop on the basis of an innate emotional program that coordinates the different facets of the organism response (e.g., expressive, physiological, or behavioural responses) when confronted with an identified threat. Some theorists have proposed that basic emotions are rather short-lived, which distinguishes them from mood [5]. In this perspective, panic has been conceived as a paroxystic fear, that is, a full-blown fear expressed and experienced at the maximum of its possible intensity [1]. 

In human psychology, anxiety is often thought of as a secondary emotion, this is, as an emotion in response to a primary emotional reaction [1, 7]. Anxiety would be the fearful reaction to another emotion, be it, for instance, fear or anger. For example, in panic disorder, anxiety is conceived as the fear of the panic (fear) response. In anxiety, the stressor is not always clearly identified, in contrast to what happens in fear. Such definition implies that anxiety requires more cognitive capacities than fear. Anxiety necessitates the capability to hold a representation of an emotional state and to react to it. This representation might be rudimentary, for instance, the reactivation of the emotional somatic state (e.g. the concept of somatic marker [8]), but it constitutes a necessary condition to anxiety. This implies that anxiety should appear in higher species when compared to fear. This definition parallels the conceptual construct that has been proposed by Robert and Caroline Blanchard in animal research. Indeed, these authors hold that the key factor distinguishing fear from anxiety is the immediacy (or certainty) versus the potentiality (uncertainty) of the threat and they define anxiety as an anticipatory fear [9]. 

Fear and anxiety are complex phenomena that articulate different components. For example, when confronted with a danger, a subject may display a specific response that includes a behavioural component (e.g., flight), a physiological one (e.g., increase in heart rate), and an expressive one (specific vocalization or facial expression). 

As an emotion, anxiety supposedly orients the organism toward a specific type of interaction with its environment [10] and thus mobilizes the entirety of the organism resources. In this perspective, anxiety comprises several elements that constitute an emotion. These elements can be categorized as, on the one hand, the different facets of the emotional response, and on the other hand, the different logistic systems of the organism that provide the biological and neuronal supports to allow for these responses. In the next sections, we will present these different elements for the human species, and assess their presence across the phylum. By taking this perspective, we, by no means, imply that humans should be considered as the most accomplished species that would subsume all the evolutionary gain of other species that would be located lower in the phylum. Rather, our perspective is a pragmatic one, taking as standard the species that we know best; both from direct experience, and from accumulated scientific work on emotion. We however hypothesize that, as suggested by several emotion theorists, there might be a trend to a complexity gain when going from species situated at a low level in the phylum (protozoan or some invertebrates) to species situated at a higher phylogenetic level. This paper may thus provide a heuristic approach, indicating which aspects of the emotion phenomenon are the most relevant in a phylogenetic perspective.

## 3. THE FACETS OF ANXIETY AS AN EMOTIONAL RESPONSE

### 3.1. Action tendencies

In this section, we will present the different facets that constitute an emotion, focussing on fear, in the perspective that anxiety is the fear of an emotional state.

Emotions have been conceived as action tendencies [10] resulting from a specific appraisal of the situation. Appraisal is the process by which an emotional meaning is attributed to a situation. Appraisal does not necessarily imply complex cognitive processes; it may consist in a very rudimentary innate detection of an unconditioned stimulus. In this perspective, individuals would constantly appraise external and internal stimuli in terms of their relevance for the organism and in terms of the behavioral reactions that may be required as a response to those stimuli [11].

When a relevant stimulus is identified, physiological, motor, and expressive response systems are activated, which constitutes the action tendency. This concept refers to the inner dispositions (or their absence) of performing certain actions or achieving certain relational changes with the environment. In other words, an action tendency is the activation of a behavioural plan aiming at changing the individual-environment relation. Impulses of “moving towards,” “moving away,” and “moving against” are examples of action tendencies [12]. The various types of action tendencies depend upon the biological constitution of the organism. Hence the phylogeny would bring along a number of such action tendencies, organizing, for instance, defence and attack, protection, attention orientation, or inhibition. According to Frijda [10, page 409], the basic emotions in human, such as those proposed by Darwin [13], Tomkins [14], or Izard [6], are the reflection of these action tendencies inherited from the phylogeny. Of course, as it is the case for facial expression, these innate programs could be modulated and accommodated through learning.

Such actions tendencies can be found in a ubiquitous manner across the phylum. For example, avoidance of danger and flight has been observed in protozoan such as paramecia [15, 16], which suggests that a central nervous system is not necessary as to the expression of such behaviours. In almost all invertebrates such as molluscs or arthropods (insects or crustaceans), specific behavioural responses can be observed when a subject is faced by threat, including withdrawal from the danger, absence of movement, and reduction of nondefensive behaviours. For example, *Aplysia californica*, a gastropod mollusc, is able to react to a threatening stimulus by escaping locomotion [17]. Further, lack of movement can be observed in several insect species when faced by danger [18]. Finally, when confronted with threat, *Aplysia* displays a reduction of nondefensive behaviour such as feeding [17]. All these behaviours are remarkably conserved through the phylum and they are also observed in vertebrates including reptiles, fish, birds, and mammals.

It is to be noted that, in humans, action tendencies are not necessarily immediately enacted [10]. They would constitute a *preparation* of the organism to react in a certain way, but the actual reaction would depend upon a sufficient activation of the action tendency. Thus, some species would benefit from a buffer between the activation of a response mode and its actual enactment. This is found in many species, including invertebrates. For example, it has been shown that environmental disturbances such as light, a drop of water, or a pebble dropped in the aquarium induce a modification of the ventilatory rate and the heart rate in crustaceans such as crayfish. These modifications occur before the animal would undergo behavioural activity. Further, in case the intensity of the fearful stimulus is low, the animal will not display any behavioural modification. These physiological modifications have been interpreted as indicative of an animal's intention for body movement before physical movement occurs [19]. 

### 3.2. The appraisal component

 Regarding the appraisal or evaluation component, Scherer [20] proposes a specific hierarchy of mechanisms for the on-going appraisal of the environment and he presents specific hypotheses regarding the pattern of evaluative meaning that should precede particular emotional states. His theory is particularly interesting in the present context as specific predictions are made regarding the phylogenetic trend. 

In human, specific emotions would be brought into play by the operations of a series of five stimulus evaluation checks (SECs). These checks are performed rapidly by mechanisms that continually scan the objects in the perceptual field, with different patterns or outcomes of the check process seen as giving rise to different emotions. Based on logical, phylogenic, and ontological arguments, Scherer [11] postulates that the SEC sequence order is fixed, with the more fundamental SECs in terms of adaptation coming first. The first SECs could be found in very simple organisms without neocortical processing capacities [11]. Thus, Scherer [11, page 41] postulates that “rudimentary forms of the novelty, intrinsic pleasantness, and even the need/goal significance checks are ‘hard-wired’,” suggesting that they can be genetically transmitted, and thus conserved by evolution. 

The first SEC, “novelty check,” looks for potential changes in the pattern of the situation. The orientation reflex is one of its consequences. Scherer [11, page 306] states that, in human, the novelty SEC is at least partly independent of higher cortical functions and may result from preprocessing in the brain stem or limbic structures. In other species, the novelty check might be totally genetically determined and independent of any neural system. This ability exists in an ubiquitous way across the phylum, including in protozoan and invertebrates. It can for example be detected using habituation: when an animal has been exposed repeatedly to a new stimulation and has established that it is inconsequential, it is able to ignore it, a phenomenon termed as habituation. Habituation has been demonstrated in all organisms across phylogeny including single-celled protozoa [10], invertebrates such as nematode ancestral worm *Caenorhabditis elegans* (which is much studied by neurobiologists because it has a fully mapped nervous system comprising exactly 302 neurons) [22], insects such as fruit flies [23], or mollusc such as *Aplysia* [24], and vertebrates such as fish [25], rats[26], or humans [27].

The second SEC is the “intrinsic pleasantness check.” On the basis of innate feature detectors or of learned associations, this second SEC evaluates the pleasantness of the stimulus or situation, hence determining approach or avoidance [11]. Scherer [11] stresses that this check has to do with the inherent pleasantness of a stimulus, and that it is not dependent on stimulus relevance to the goals of the organism. Again, in human, this SEC would be partly independent of cortical structures and some of its processes might take place in the amygdala. In other species, this check might be totally determined by automatic processes. If an animal is able to display either approach or avoidance of a stimulus present in its surrounding, or to undergo appetitive or aversive learning, one may conclude that it possesses the ability to do this check. According to some authors, the approach-avoidance distinction is also applicable to organisms as simple as the protozoa amoeba. In this case, approach and avoidance behaviours are extremely basic [28, page 2]. For example, in amoeba, a weak light will stimulate a movement in that direction, whereas an intense light will elicit a withdrawal from the light source. Approach and avoidance can also be observed in more sophisticated invertebrates including ancestral worms and insects. For example, the nematode *Caenorhabditis elegans* is able to display preferences for some stimuli over others [29], to avoid noxious chemicals, high osmolarities, acidic pH, and noxious mechanical stimuli [30], and to display aversive learning [31]. Insects such as drosophila display appetitive as well as aversive conditioning [32]. In fact, Schneirla [28] argued that organisms at all levels of complexity, ranging from protozoan to higher vertebrates, possess what he termed A-type (approach-type) mechanisms, facilitating food-getting, shelter-getting, and mating, and W-type (withdrawal-type) mechanisms, enabling defence, huddling, flight, and protection in general. He proposed that the sophistication of these mechanisms varies considerably across the phylum, those of protozoa and invertebrates being rudimentary and rigid, and those of higher organisms being more complex and flexible (see also [33, 34]). These two reactions have survival value, as they move the organism toward beneficial stimuli and away from harmful stimuli [35, page 7] and are therefore conserved from protozoan to higher vertebrates.

Goals and needs of the organism come into play in the third SEC, the “goal/need conductiveness check.” It examines the extent to which the introduction of the detected stimulus or event will advance or hinder the attainment of a specific goal or the satisfaction of a need. The goal/need conductiveness check is divided into three subchecks: the relevance subcheck that examines the relevance of the stimulus or event for important goals/needs of the organism, the expectation subcheck that determines the stimulus consistency with the state expected at this point in the goals/needs sequence, and the conductiveness subcheck that determines if the stimulus is conducive or obstructive to the respective goals or needs. This check can also be entirely genetically determined.

If a given animal is able to display specific behaviour to escape stimuli that are incompatible with its survival such as predators or high temperatures, one can consider that it has this capacity. This can be seen in almost all invertebrate species. For example, nonsegmented worms such as nematodes escape when exposed to temperature above 33°C (for a review on nematodes see [36]). Other invertebrate have specific behaviours to escape predators: cuttlefish can bury into the sand to hide themselves from predators [37], grasshopper may display immobility when confronted with a frog [38] as well as beetles when attacked by spiders [39]. This kind of behaviour is also observed in protozoan. For example, ciliated protozoans such as *Euplotes* are able to change their morphology [40] and behaviour [41] in response to predators [42]. Of course, these data do not enable to distinguish the capabilities of these species regarding the different subchecks of this appraisal component; such a detailed analysis being beyond the scope of this review.

So, this third SEC has not been altered significantly through evolution, as it is described in invertebrates, and even protozoan such as ciliates as well as higher vertebrates. This is probably related to the fact that it is essential to the survival of the different species. One should note that, at the methodological level, the distinction between the second (valence) and third (goal conductiveness) SEC might not be possible to operate in lower-order species. Beyond this methodological limitation, an alternative hypothesis should be considered: this distinction might not be relevant. In species low on the phylogenetic scale, these two SECs might not be differentiated. Their distinction would only appear in higher-order species.

These three first checks have also been studied in an extensive and systematic way in some mammals, such as for example lambs [43, 44]. These species display specific behavioural and physiological pattern of response when subjected to environmental challenges characterized either by novelty, by intrinsic pleasantness, or by having need/goal significance.

The fourth SEC, the “coping potential check,” determines the cause of the event, and the capacity of the organism to control it or to confront it, or to adjust to the final outcome. If a species is able to react in a different way in function of the predictability/controllability of a signal, one may claim that it has this ability. To our knowledge, no study has been published addressing the presence of such processes in ancestral worms or protozoan. Ancestral worms such as nematodes possess the ability to assess the rhythmicity of some events; this is necessary but probably not sufficient to possess the ability to react in function of the uncontrollability of an event. Such changes of behaviour in function of the controllability of a stimulus have been described in mammals such as dogs by Overmier and Seligman [45]. Indeed, in dogs, prior inescapable electric foot shock interferes with later escape/avoidance learning in which shock is the negative reinforcer, a process termed as learned helplessness. One may claim that if a species displays learned helplessness, it might react in a different way depending upon the predictability/controllability of the situation. Learned helplessness has been described in various mammals including dogs, rats, mice, cats, and sheep [45–53] but also in lower vertebrates such as fish [54–56]. Further, insects such as cockroach also exhibit a failure to escape shock when possible to do so following nonescapable/uncontrollable shocks [57–59] in a similar way as vertebrates displaying learned helplessness. Therefore, one may claim that the “coping potential check” may be present in several species across the animal kingdom, including all vertebrates and some invertebrates such as insects. However, no evidence exists in more rudimentary invertebrates such as worms.

Finally, the last SEC, the “norm/self compatibility check,” evaluates the congruence of the event with the social and individual norms and standards such as mental prescriptions, self-concept, and self-ideal. This check needs the presence of cultural transmission. The presence of culture in animals such as nonhuman primates is still debated. Some authors claim that “proto-cultures” or “traditions” (defined as “long-lasting behavioral practices shared among members of a group partly via social learning,” see [60]) can be observed in animals. This for example has been first described in the early fifties [61] in a group of Japanese macaques (*Macaca fuscata),* a species displaying acquisition of innovative behaviours, such as potato and wheat-washing, first displayed by a young female and then transmitted to social partners as well as to successive generations [62]. In chimpanzees (*Pan troglodytes),* behavioural variants (traditions) have been described in different communities, such as differences in tool usage, grooming and courtship behaviours [63]. However, all authors would not agree that these traditions can correspond to the cultural transmission seen in humans. According to Donald [64], humans have three cognitive processes (mimetic skill, language, and external symbols) not available to other primates and enabling such a transmission. Others propose that sophisticated forms of imitation that are only described in humans are necessary for cultural transmission [65]. Similarly, some argue that culture is a uniquely human form of social learning, requiring imitative learning, instructed learning (teaching), and collaborative learning, three social-cognitive processes emerging in human ontogeny [66].

The pattern of the outcome of the different SECs determines a particular emotional meaning and directly activates the corresponding action tendency. In human anxiety, the central features are that aspects of the situation are evaluated as intrinsically negative (intrinsic pleasantness check), as threatening important goals of the organism (e.g., survival, or social acceptation in a gregarious species) (goal/need conductiveness check), and as unpredictable or uncontrollable (coping potential check). Thus, to experience full-blown anxiety, a species would need to have the capacity for the first four SECs defined by Scherer's theory. As previously shown, all these four checks seem to be present in an ubiquitous manner in the different phyla, from invertebrates such as insects to lower vertebrates (fish) and mammals and even, for some of them, in unicellular organisms such as protozoan. Therefore, according to this theoretical frame, some rudimentary form of anxiety may be present from invertebrates to humans. However, as we will see, the level of sophistication, as well as of awareness of these evaluations and of the resulting experience vary tremendously from species to species, according to their cognitive capabilities.

### 3.3. The physiological component

As action tendencies, emotion and anxiety recruit all the logistic capacities of the organism. The physiological systems are activated in order to support the actions and transactions with the environment called for by the emotional situation. In humans, many physiological and endocrine responses have been observed in emotion and in anxiety in particular. There is still a debate regarding whether specific emotions (and anxiety can be considered as such) have unique physiological characteristics. Despite a century long tradition of physiological research in human emotion, no definite conclusion has been reached yet [67]. Physiological responses in human emotions seem to result from a complex interaction between the demand of the situation, personality characteristics, and the type of regulation strategies used in that situation [68].

Regarding fear and anxiety, meta-analyses of the literature have documented marked changes in most peripheral responses: cardiovascular changes, respiratory changes, muscles tonicity changes, or skin temperature changes when compared to neutral states [67]. These changes are driven by the autonomic nervous system. These changes, however, are not that different from other intense emotions such as anger, with the exception that anger produces more elevated diastolic blood pressure.

Most of these reactions are present in rodents such as rats, and they can vary as a function of the behavioural response that the subject may display. For example, a flight response can occur in response to threat that is associated with increased blood pressure and tachycardia, enhanced cardiac output and respiration, increased cerebral perfusion and redistribution of blood flow to increase limb circulation [69–72]. Some aspects of these responses are also observed in lower vertebrates such as fishes. Indeed, salmons show flight associated with increased heart rate when confronted with a simulated predator attack [73]. Other components, such as variations in skin temperature or skin conductance are difficult to measure without stressing the animals, so that the few empirical studies that assessed these modifications were only done in mammals using radiotelemetry. For example, a decrease in skin temperature following alerting stimuli has been shown in monkeys in different parts of the body including the nose, nasal mucosa, ears, hands, feet, and tail [74]. Such temperature variations according to fear or anxiety are logically absent in lower vertebrates, which are poikilothermic. Other aspects of the human physiological response to threat are not present in lower vertebrates. For example, fishes, amphibians, and reptiles do not have dilatator musculature innervating the iris so that they may not exhibit mydriasis.

Even if not possessing an autonomic nervous system similar to the one enabling the physiological response to danger seen in vertebrates, invertebrates need the same rapid cardiovascular and respiratory regulation to be primed for the defensive behaviours they exhibit toward threatening stimuli. Indeed, such modifications provide the organism with the metabolic/energetic resources that will be necessary to deal with environmental challenges. Are such physiological responses observed in invertebrates when confronted with danger? Are they associated with the behavioural response? In crustaceans, perception of changes in the surroundings of the animal can induce modifications of some physiological variables such as heart rate and ventilatory rate [19]. This is also seen in molluscs such as cephalopods. For example, octopus displays cardiac arrests when exposed to a stressful situation [75]. Thus, the physiological responses observed in some invertebrates such as crustaceans or molluscs faced by threatening stimuli are very close to the responses of vertebrates mediated by the autonomic nervous system [76]. In other invertebrates such as insects, the energy necessary to cope with threat is provided to the organism by other means. For example, in insects, the blood flow to the different tissues is not regulated by an increase of the heart rate. Indeed, insects have an open circulatory system that differs from the closed circulatory system (in which blood is always contained within vessels) found in vertebrates. In an open system, blood (termed as hemolymph) flows freely within the body and establishes direct contact with all internal tissues. In case of danger, hemolymph delivery to the tissue is directly increased, without a modification of heart rate. However, even if modifications in heart rate have not been documented in fear-challenging situations, behavioural activity induces modification in heart rate (C. Lazzari, personal communication). As fear is associated with modification of activity, it can thus be that it is related to heart rate modifications.

Thus, it is possible that the representation of the body changes occurring during danger may be very different depending on the species: mammals may perceive environmental-induced changes driven by the autonomic nervous system in their body and including modifications in heart and ventilatory rate, in skin temperature, and mydriasis, lower vertebrates (amphibians, reptiles, fish) and some invertebrates (crustaceans, molluscs) may exhibit modified heart and ventilatory rate without changes in temperature or mydriasis.

### 3.4. The expressive component

Emotions are not only inner states. They are also communicated to the environment, as they convey the behavioural intend of the individual. In human, the expressive component has certainly been the most studied, at least for facial expression. A series of studies has demonstrated innate and cross-cultural aspects of emotional facial expressions in humans. However, these innate facial displays are modulated by a set of cultural and display rules [77, 78]. The gist of this literature is that the nonverbal communication of emotion serves very important functions of regulation, both within the species and cross-species. It is conceived of, primarily, as a social process.

While much work has been devoted to the facial display of fear, the literature in human is almost silent regarding a facial expression that would be specific to anxiety. Most scholars do not distinguish facial expression between these two states [6, 79]. Similarly, the studies that have investigated modulations of prosody during emotional states did not distinguish fear from anxiety [80, 81]. Yet, emotional prosody in humans has clear phylogenetic roots that have been traced back to primates [82]. This point will be developed in the following paragraphs.

An interesting phenomenon for emotion regulation, known as facial feedback, has been documented in humans [78, 79]. A wealth of research has established that holding a certain nonverbal expression was generating or reinforcing the corresponding affect. Thus holding a nonverbal expression of anxiety generates and intensifies this emotion. Phenomenon of contagion via mimicry has also been documented [83–85].

In humans, some studies have documented that different emotions were expressed by different postures (e.g., [86]). Further, Stepper and Strack [87] have documented that manipulating posture has an impact on the emotional subjective feeling state and affects later judgment of valenced material. Further, there is some evidence that body odours are modulated by emotion, including fear and anxiety. For instance, Chen and Haviland-Jones [88] have collected underarm odours on gaze pads in human subjects exposed to a joyful or a frightening movie. The authors have observed that, on the only basis of the collected odours, human participants could detect above chance level the emotion induced.

In animals too, emotional state can be communicated to the environment by specific signals, including facial, postural, vocal, or chemical ones. Further, other kind of expressive components are also documented, including more specific ones such as camouflaging.

Modification of facial expression in relationship to emotions can be seen only in species having a well-developed facial musculature. Facial musculature is highly conserved across primates [89], the one of chimpanzee being almost identical to that of humans [90]. Indeed, in this species, specific facial expressions have been described in response to danger such as fear grin. However, even if some spare evidence indicates that some mammals such as rats are able to display some specific facial expression to the affective aspects of taste [91], the facial musculature of nonprimate mammals is undeveloped or nonexistent [89, 92, 93] and may not allow more specific facial expressions.

Postural changes have been extensively described in higher vertebrates confronted with danger. For example, rodent may display a posture characterized by immobility, flattening of the ears, piloerection, and marked mydriasis. Indeed, specific postures have been repeatedly seen in vertebrates in emotional situations: they have been nicely illustrated by Darwin [13].

Specific vocalizations to threat have also been documented across the phylum. For example, vervet monkeys emit specific alarm calls to different predators such as leopards, eagles, or pythons [94]. Variation in alarm calls with the type of predator has also been described in rodents such as gerbils [95]. In other species, these calls are less sophisticated as they may indicate the presence of a danger to congeners, without giving more information on the precise nature of the threat. Specific vocalizations to danger have been described in birds [96], but also in amphibians (e.g., crocodiles [97]) and fish [98]; they are thus present across the vertebrate phylum. Further, such calls have also been described in invertebrates such as insects. For example, Wyttenbach et al. [99] showed that field crickets emit ultrasonic signals in the 25–80 kHz range when confronted with predators, inducing escape behaviour in other crickets. However, all signals emitted by these crickets do not elicit the same response: when they produce signals in the 4-5 kHz, conspecifics approach, indicating the specificity of these alarm calls. So, vocal expressions related to danger can be seen in vertebrates as well as in invertebrates.

The use of pheromones to alert conspecifics of the presence of a danger is common in many animal species. For example, in the presence of an intruder, several species of social hymenoptera secrete pheromones that cause defensive behaviour among conspecifics [100]. Such reactions can be found in vertebrates as well. For example, carnivorous mammals of the Mustelidae family use anal scent glands to produce olfactory warning, often repellents signals [101]. Fear may be communicated by odours in mice and rats as well [102]. Such reaction can also be documented in nonhuman primates. Indeed, it has been shown that the genital scent glands of two prosimian primates are involved in producing a fear scent [103].

Camouflaging can be considered as a form of behaviour intermediate between emotional expression and coping with the situation. Indeed, it often appears when a species is confronted with a danger such as a predator. The most common form of it involves the modification of the visual appearance, but calls, songs, and scents can also be changed. Different strategies of camouflaging have been described, such as crypsis, aposematism, Müllerian mimicry, and Batesian mimicry. Crypsis enables to minimize the signal to noise ratio, thus rendering the detection of the subject very difficult for a predator. It generally consists in matching colours and patterns between an animal and its background [104–106]. It is very common in invertebrates such as arthropods (e.g., in insects) or molluscs (e.g., in cephalopods) as well as in some vertebrates such as fishes, amphibians, reptiles, and birds. For example, the day octopus (*Octopus cyanea*), which forage on coral reefs, produce colour patterns capable of instantaneous matching to backgrounds from sand and reef rubble, through to spiked corals and seaweeds. More rarely, this kind of defence strategy can also be seen in mammals. For example, in the rock pocket mice *Chaetodipus intermedius* and in the deer mouse *Peromyscus maniculatus,* variation in coat colour, as a function of the colour of rock substrate, has been documented. This strategy is adaptive, providing the mice cryptic protection against predators [107]. The other camouflaging strategies (aposematism, Müllerian mimicry, and Batesian mimicry) are based on a maximization of the signal to noise ratio. Aposematism consists in displaying warning signals (e.g., conspicuous coloration) informing a potential predator that the prey is toxic or unpalatable. It exists in many invertebrates, but also in fishes, amphibians, snakes, and birds [108]. Batesian mimicry is a form of mimicry in which an innocuous unprotected species closely resembles a noxious model species. Hoverflies that resemble bees or wasps are an example. This can involve the coloration pattern as well as some aspects of the animal's posture. For example, the Indo-Malaysian octopus can adopt a colour and a posture mimicking a poisonous sea snake. In Mullerian mimicry, two or more equally poisonous species share an identical colour pattern, thereby reinforcing the warning each gives to predators. In some cases, dynamic camouflage can be observed: some insects imitate the movements of branches or leaves in their surrounding.

### 3.5. Cognitive mode

In human psychology, extensive research has documented that emotion in general, and anxiety in particular, are accompanied by specific cognitive response. Threat and anxiety have been shown to powerfully affect attention allocation. Laboratory studies have documented that threatening stimuli automatically attract attention, even during subliminal exposure (very rapid presentation that cannot be consciously perceived) (for a review, see [109]). In people suffering from chronic anxiety, this pattern would be even more pronounced and aggravated by a poor capacity to disengage attention from threat. In fact, most models of human anxiety (e.g., [110]) consider that an attentional bias toward threat is an essential component of anxiety, especially of dysfunctional anxiety.

Attention bias toward anxiogenic stimuli has rarely been examined as such in nonhuman animal species. However, different phenomena have been described in animals that can be interpreted within this frame, including fear-potentiated startle, increased cognitive performance in stressful situations, anxiety-induced increased attention toward negative stimuli and a bias for threat cues in anxious mice.

Fear-potentiated startle corresponds to an increase of the amplitude of the acoustic startle response in the presence of a cue previously paired with a shock. It has been described in rhesus monkeys [111] but also in rodents such as rats [112] or mice [113]. To our knowledge, fear-potentiated startle has not been examined in nonmammalian vertebrates such as birds or fishes.

Another phenomenon that has been widely documented is the increased mnesic performance observed in anxiogenic situations: this is generally attributed to the fact that anxiogenic situations increase attention, thus increasing mnesic encoding. This facilitation has been repeatedly observed in rodents such as mice but also in birds. The processes used to increase anxiety include pharmacological manipulations, lesions studies, maternal separation in pups, genetic invalidation, and strain variations. For example, a principal component analysis showed that, in mice, higher emotional memory performance is related to heightened state anxiety [114]. Further, Venault et al. [115] showed that, in rodents but also in chickens, anxiogenic compounds increased memory in three different tasks, while anxiolytic drugs elicited opposite effects. However, this association is probably not causal, as *β*-CCT, a selective benzodiazepine receptor antagonist, blocks the antianxiety but not the amnesic action of benzodiazepines in mice [116], suggesting that the anxiolytic and the amnesic effects of these compounds are independent. In mice, a multiple regression analyses also revealed a relationship between attention toward salient stressful stimuli in a conditioned task and sensitivity to stress [117], suggesting that attention toward negative events may contribute to the response in stressful situations. Finally, when mice characterized by heightened anxiety-like behaviour are subjected to a fear conditioning protocol including a fully conditioned stimulus (a tone always followed by a shock) and a partial conditioned stimulus (a light, only partially related to the shock), normal mice discriminate between the partial and the full conditioned stimulus, while the anxious mice show the same response to the two stimuli [118]. This phenomenon has been interpreted as a bias for threat cues.

Most of these studies suggesting an attentional bias toward threat in anxious animals have been conducted in mammals, specially rodents, the sole exception being the pharmacological studies that were also conducted in birds. Even if the absence of such studies does by no ways mean that such processes do not exist in lower vertebrates, it suggests that it is at least difficult to assess in fish, amphibians, or reptiles. A reason for that could be that this facilitation does not occur in that species, but this remains to be confirmed by experimental studies.

### 3.6. The subjective feeling component

In the human literature, an important component of emotion is of phenomenological nature: the subjective feeling state. It reflects the notion that, when emotional, the individuals feel in a different state that colours their perception of the world and of themselves. Most authors agree that the subjective feeling component results from the global perception by the individual of the changes operating in the different emotion facets [119]. There is also a consensus on the fact that the subjective feeling state can vary in terms of awareness. For instance, Lane [120] has identified several levels of awareness of emotion, from a diffuse sense of bodily changes, to the reflexive awareness of observing oneself in an emotional state. These different levels of awareness are supported by different brain structures. They supposedly progressively appear during the ontogenesis, with the highest level of awareness fully mastered only at adolescence.

Reflexive emotional awareness is particularly relevant for emotion regulation in general and anxiety in particular. This capacity enables humans, not only to be reflexively aware of their on-going experiences, but also to reactivate past experiences, or to imagine future ones [121]. The capacity for self-consciousness, labelled autonoetic consciousness by Tulving [122], is the central element that allows remembering specific past experiences (i.e., episodic memory) as well as for imagining what future experience would feel like. As a form of anxiety consists in an apprehension for a future emotion (e.g., fear or anger), it implies the capacity to envision what a future experience would feel like. Hence, possessing autonoetic awareness capacities opens many avenues for anxiety to develop. For instance, for a student, the capacity to imagine a future examination creates a source of anxiety. On the contrary , it has been observed that people who, because of cerebral damage in the frontal and prefrontal regions, lack any autonoetic capacities (for a review, see [121]) are unable to experience any anxiety.

The capacity for autonoetic consciousness is one of the last cognitive features to develop in the human ontogeny. Its first manifestation in terms of reflexive capacities to one's own experience appears around 4 years of age and it is believed to be only fully developed around 14 years of age [121]. To date, the evidence for autonoetic consciousness in non-human primates is still the object of a debate [123]. This debate is further fuelled by the fact that the exact cognitive processes leading to autonoetic awareness are still to be identified. However, the brain regions involved, as well as the important cognitive resources required, strongly suggest an important involvement of executive processes.

As autonoetic consciousness is a key feature of episodic memory [122], the development of episodic memory across species might shed some light on the birth of autonoetic consciousness along the phylum. Several reviews of this question have been proposed (e.g., [123, 124]). However, it should be stressed that autonoetic consciousness does not only imply the capacities to remember “what, when, and where” a specific event occurred. This latter capacity seems to be acquired early in the phylum, as it is already mastered by birds [123]. Rather, autonoetic consciousness also implies the capacity of representing oneself as the subject of the experience remembered. This latter facet implies self-awareness. This capacity seems to appear very late in the phylum. According to Gallup et al. [125], self-awareness can be reflected by self-recognition and by the ability to infer mental states in others. Indeed, according to these authors, if a subject is able to have a representation of itself, it may possess the ability to identify itself (self-recognition) and to use its own experience to infer comparable experience in others (a process termed as mental state attribution or theory of mind). Therefore, self-recognition and mental state attribution could be heuristic indicators of self-awareness. Gallup [126] developed a paradigm enabling to test self-recognition in great apes: the capacity to interpret one's own reflection in a mirror. It has been shown that mirror self-recognition exists in chimpanzees [126, 127], but also in other great apes including orangutans and bonobos [128, 129]. Interestingly, this capacity has not been seen in some great apes such as gorillas [128, 130] or in monkeys such as macaques [126]. Further, self-recognition has also been shown in great apes using other paradigms [131]; however, it was never observed in other nonhuman primates, suggesting a phylogenetic gap for this process between great apes and other nonhuman primates such as macaques.

It should however be noticed here that the assumption that great apes are able of self-recognition of their image in a mirror has been questioned by some authors, and is still matter of controversy. Indeed, according to some authors (see, e.g., [132]), the behaviour of these primates when faced with a mirror could instead have occurred by chance or result from experimental artefacts. On the other hand, evidence of mental state attribution in animals is still matter of controversy. It seems that this process appears very late in the phylum. Scarce evidence indicates that chimpanzee may be able to take into account what other chimpanzee can or cannot see [133]; however, this question remains a contentious issue [132]. So, some controversial evidence indicates that great apes such as chimpanzees, bonobos, and orangutans may possess some abilities such as self-recognition, that reflect self-awareness, a process necessary for autonoetic consciousness. However, at this point, prudence is necessary because this by no means indicates that they possess autonoetic consciousness. This just means that they have some abilities enabling this kind of consciousness.

## 4. THE LOGISTIC SYSTEMS OF THE ORGANISM SUPPORTING THE ANXIETY RESPONSE

In humans, the anxiety response is supported by several biological systems, including neurotransmitters such as biogenic amines, stress hormones, activity driven by the autonomic nervous system, and changes within specific brain areas. Are these different features present at all levels of the phylum?

Fear triggers the release of various biogenic amines, including the catecholamines adrenaline, noradrenalin, octopamine, and dopamine and the indolamine serotonin. Adrenaline, noradrenalin, and dopamine have been described in all vertebrates, with some variations that have been suggested to be related to an evolutive trend [134]. Indeed, high noradrenalin/adrenaline ratio appears to be characteristic of more primitive vertebrates while a lower ratio occurs in tetrapods and mammalian adults. In invertebrates, all catecholamines have been detected in several insects, but also in scorpions as well as in gastropods and cephalopods [135]. Serotonin has also been detected in several invertebrates including arthropods such as scorpions, insects, or crustaceans, or molluscs such as cephalopods [136–140]. Are these biogenic amines released under stressful situation similar to the ones triggering fear and/or anxiety? This seems to be the case. For example, stress elicits an increase in noradrenalin and dopamine in oysters: this response occurs rapidly and its intensity is correlated with the intensity of the stress [141]. Consequently, one may claim that there are only small variations across the phylum as to the biogenic amines.

Fear and anxiety also produce some specific hormonal release, related to the activation of the hypothalamic-pituitary-adrenal (HPA) axis, including a release of several stress hormones such as corticotropic-releasing hormone (CRH), adrenocorticotropic hormone (ACTH), and glucocorticoids. Stress hormones seem also highly conserved across the animal kingdom. Indeed, CRH has been described in various mammals but also in birds such as pigeons and quails, frogs, and several fish species (elasmobranch fish, teleosts, goldfish, salmons, eel). Such molecules are not only found in vertebrates. Indeed, CRH-like molecules have been reported in some invertebrates including in the nervous system of the annelid *Dendrobaena subrubicunda*, the insect *Periplaneta americana*, and the mollusc *Planorbarius corneus* (for a review, see [142]). ACTH release from hypothalamic centres has been observed in birds, amphibians, and teleost fish. With regard to invertebrates, ACTH-like compounds are found in the nervous system of various molluscs and insects, but also in the protozoan *Tetrahymena pyriformis* (for a review, see [142]). Therefore, this compound or its functional equivalent is present at quasi all levels of the phyla. In mammals, glucocorticoids such as corticosterone or cortisol are released by the adrenals, a gland consisting of an outer part (the adrenal cortex) and an inner part (the adrenal medulla). Nonmammalian vertebrates lack the typical anatomical adrenal gland of mammals, but they are equipped with cells resembling mammalian cells of the adrenal cortex. Corticosterone has been detected in some birds such as chickens or ducks, reptilians, amphibians, and fish but also in some invertebrates, particularly insects (for a review, see [142]). So, again, there are very few variations in stress hormones across the phylum.

The phylogeny of the autonomic nervous system has been extensively studied by Nilsson [143, 144]. It appears that this system is more or less the same in all vertebrate species, with the exception of the lower fishes (cyclostomes) that do not have the double cardiac innervation (noradrenergic and cholinergic) that all the other vertebrate species have (from higher fishes to mammals). Invertebrates do not have autonomic nervous system as vertebrates; however, past work undertaken by comparative neuroanatomists such as Zavarzin [145] drew similarities between the sympathetic nervous system of vertebrates and the unpaired nerves of insects.

Another important system supporting the human anxiety response is the facial musculature, enabling the facial expression of emotions. Such musculature is not present in invertebrates having an external skeleton, such as insects or bivalves. In nonmammalian vertebrates, this musculature is very rudimentary, enabling only opening and closing of the apertures such as mouth, eyes, and nostrils [146, 147]. Greater mobility of the lips can be seen in mammals, probably because this may facilitate suckling [148]. In primates, facial musculature gains in complexity as specific muscles appear that enable emotional facial expression (e.g., zygomaticus major, zygomaticus minor, levator labii superioris, depressor angulioris, depressor labii inferioris, and risorius) [148]. The facial musculature is innervated by neurons originating from the craniofacial motor nuclei (VII) of the brain stem. According to Sherwood et al. [146], a basic pattern of muscle representation in the craniofacial motor nuclei is strongly conserved across mammals. However, counting of the number of neurons in these areas shows that hominids (great apes and humans) have 24% more facial neurons than predicted from their medulla size, indicating a larger development of this structure in great apes and humans. Further, in old world anthropoid primates, cortical neurons originating in the motor cortex and projecting directly to cranial nerve motoneurons have been described: there is no evidence of such direct projections in other mammals [146, 147]. These projections may enhance volitional control over facial expression. So, facial musculature and the structure that control it are mostly described in higher primates such as great apes and humans.

Several functional neuroimaging studies have investigated the brain structure whose activity is modified during fearful experience. For example, activation of the amygdala has been observed during acquisition of conditioned fear [149]. This involvement of the amygdala has then been largely confirmed [150]. Further, during fear conditioning, an activation of the anterior cingulate cortex is also observed and, in case of trace fear conditioning, an additional activation of the hippocampus has been documented [151]. These authors suggest that the hippocampus may enable the storage of the spatiotemporal aspects of the fear experience, while the anterior cingulate cortex may permit to drive attentional resources toward the stimulus and to anticipate the occurrence of the fearful stimulus. Other studies focused on brain activation during anticipation of fear. They showed that during anticipation, subjects report fear experience associated with activation of the physiological variables related to fear. Further, these studies revealed that during anticipation, there was an activation of the prefrontal cortex [152] (particularly of the orbitofrontal cortex [153]), of the temporal area [153, 154], and of the insulae [153]. Finally, when subjects are requested to try to self-generate emotions by re-experiencing past events, they show a decreased activation of the hypothalamus, of the posterior cingulate cortex, and of the orbitofrontal cortex and an increased activity in secondary somatosensorial cortices, in the insulae, and in the hippocampus [155]. Interestingly, some of these modifications are observed in areas enabling the perception and the regulation of body internal states (somatosensorial areas and insulae). So, these studies show that several brain areas are engaged in humans during fear or anxiety, including subcortical ones (hypothalamus, amygdale, hippocampus) and cortical ones (prefrontal cortex, somatosensorial areas, insulae, cingulate cortex).

Is such a pattern of activation also observed in other species? How does the anatomy of these brain areas evolve across the phylum? We will answer these questions mainly focusing on vertebrates, as the nervous system is organized in a different manner in invertebrates making a comparative approach difficult.

We will first consider the phylogeny of the hypothalamus, the amygdale, and the hippocampus. The hypothalamus is a very old area and unlike most other brain structures, it has been conserved throughout phylogeny and exists in all vertebrates, including fishes. Amygdala and hippocampus have not been described as such in fishes; however, on the basis of anatomical and developmental data, it has been suggested that the fish medial and lateral regions of the telencephalic pallia might be the homologous neural structure to the mammalian amygdala and hippocampus, respectively [156–159]. Further, these areas seem to be associated with functions that are also homologous to the ones of limbic structures in higher vertebrates. Indeed, several recent studies showed that medial and lateral pallium ablation in fishes induces a deficit in fear and spatial learning, respectively [160–162]. In amphibians, similar results are obtained as the medial pallium appears to be homologous with the hippocampus of mammals [163]. Further, in these species, the basic subdivisions and connections of the amygdalar nuclei found in mammals and described [164] as structures homologous to the lateral, medial [165], and central [164] amygdala have been recently identified within the ventral part of the lateral pallium. Finally, the posterior dorsal ventricular ridge of amphibians has afferents and efferents similar to the ones of the basolateral amygdala of mammals [166]. This can also be seen in reptiles [167, 168]. In birds, the hippocampal formation is considered to be homologous to the mammalian hippocampus [169] and the posterior and medial archistriatum is considered as a homolog of the amygdala in mammals [170]. In mammals such as rodents, the amygdala as well as the hippocampus are largely equivalent to the ones of primates in their connectivity, neuroanatomy, and function. The role of hippocampus in trace and contextual fear conditioning is well established [171–174]. Further, the function of the different subdivisions of the amygdala in fear and anxiety is largely described, the lateral and central parts being involved in classical fear conditioning [175–178] and the medial nucleus being mostly related to unconditioned fear [179, 180]. So, in vertebrates, the subcortical structures implicated in fear and/or anxiety have been well conserved, the hypothalamus being present in all species, and regions homologous to the hippocampus and amygdala being present, and functionally activated during fear, in fishes. In higher vertebrates, a suborganization of these areas appears, subserving specific functions.

We now consider the phylogeny of the neocortical areas (prefrontal cortex, secondary somatosensorial areas, insulae, cingulate cortex) involved in the human anxiety. The classical view concerning the origins of the mammalian neocortex considers that it may be inexistent in nonmammalian vertebrates such as birds or reptiles. In fact, a three-layered cortex has been described in reptiles [181, 182] and some authors claim that neuronal populations homologous to the ones found in the mammalian neocortex are seen in the avian/reptilian dorsal ventricular ridge [183]. However, this view is contested. The following paragraphs discuss the presence of these areas in mammals, and mention some debates regarding their functional equivalents in birds. 

In rats, the frontal cortex is subdivided into three topologically different regions: the medial prefrontal cortex (that includes the anterior cingulate), the orbital prefrontal cortex, and the agranular insular cortex [184]. Rats have also a distinct secondary somatosensory cortex. All these areas are activated by anxiogenic stimulus (see, e.g., [185]), suggesting that they are involved in fear and anxiety. However, rats may not have exactly the same neural representation of fear as primates. Indeed, recently, some features that seem to be unique in primates have also been described. For example, it has been shown that activity within the right anterior insula correlates with conscious awareness of the bodily responses occurring during emotional states (e.g., heartbeat detection) suggesting that this area may provide a substrate for subjective feeling states [186, 187]. Interestingly, this region has a specific pattern of afferents enabling this function (e.g., the thalamocortical lamina 1 pathway) that is only developed in primates [188], suggesting that awareness of visceral changes related to emotions may only exist in primates. Further, these projections are small in macaques, and their size develops mainly in great apes. In the anterior cingulate cortex, some specific neurons termed as spindle cells have been described that are present only in humans and great apes [189]; they have been suggested to be involved in emotional self-control and problem-solving capacity [190]. Further, some specific afferents of these areas such as the ancillary thalamocortical lamina 1 pathway are also specific to primates. Within the prefrontal cortex, there is also another area that is unique in great apes and humans: Brodmann's area 10. This area may be involved “in the retrieval of memories from the individual's past experience and the capacity to plan adaptive responses” [191] which may be essential to autonoetic consciousness.

## 5. CONCLUSIONS

Table 1 presents in a simplified way a summary of the data presented in the previous sections. A clear evolutive trend appears, as the components of the emotional processes as well as the logistical systems related to their realization gain in complexity from lower to higher levels of the phylum. Further, it can be noticed that the species located higher in the phylogenetic tree, while gaining some additive abilities (cognitive bias, autonoetic consciousness), never loose the more primitive capabilities they share with the lower invertebrates. Therefore, the human anxiety may indeed be based on aspects inherited from the evolutionary history as well as on high-order cognitive processes. Table 1 clearly shows that some very rudimentary aspects of the behavioural responses are present in unicellular organisms such as protozoan and ancestral nonsegmented worms such as nematodes (novelty, pleasantness, and goal conductiveness checks, associated with a behavioural response and with the presence of stress hormones), probably indicating the high survival potential of these aspects of emotional responses in general and of anxiety in particular. In insects, the response is enriched by an additive appraisal check (coping potential), the presence of a specific emotional expression characterized by postures, vocalisations, and pheromones, and by the release of specific monoamines in response to environmental challenges. The physiological response to danger is documented in crustaceans as well as molluscs; this enables us to distinguish the pure behavioural response from action tendencies in which a modification in the physiological indicators may appear before the behavioural response occurs. The logistic systems supporting the main facets of human anxiety appear in vertebrates (the vegetative and central nervous systems). Low-order vertebrates (fish, amphibians, and even reptiles) possess an autonomic nervous system coordinating the physiological response to stressful situations. This system is associated with the hypothalamus and brain areas that are functionally homologous to subcortical areas involved in fear in higher-order species (e.g. the amygdala and the hippocampus). In birds, specific responses related to their ability to regulate body temperature appear. In mammals, a functional amygdala is present, with many subdivisions. Primates are characterized by their ability to display specific facial expressions in reaction to danger; they are associated with an important facial musculature. Finally, some very sophisticated facets of emotional processes such as autonoetic consciousness appear in conjunction with some specific connections of parts of the prefrontal areas necessary for the conscious perception of the visceral changes related to emotions, of emotional control, or of retrieval of memories from past experience.

At first sight, Table 1 reveals a striking phenomenon: many emotional processes related to anxiety can be executed even in the absence of the logistical structures that support them in humans. For instance, while insects already display a large range of emotional processes such as appraisal, action tendencies, and emotional expression, they are lacking many of the structures, especially in the vegetative and central nervous systems, that are governing these facets of anxiety in humans. This observation is even more pronounced in crustaceans and molluscs. This suggests that the processes and functions active in anxiety appear in lower-order species that have not developed the neural, chemical, or anatomical structures that support them in humans. In these lower species, functionally equivalent structures might organize these processes. Further, on the phylogenetic scale, the evolution would have developed *ad hoc* structures for more functional diversity and efficiency. This view is in line with a Lamarckian perspective on the phylogeny of anxiety.

Another remarkable point that can be seen in Table 1 is that insects possess the four SECs necessary to fear. Indeed, they have the ability to appraise the novelty, the pleasantness, the goal conductiveness, and the coping potential of a given situation. Interestingly, these abilities exist independently of other features of the anxiety response, such as the physiological response to fearful situations. These processes seem independent of the presence of specific brain areas such as limbic structures that do not exist in the insect nervous system, which suggests that they may be realized via other logistical systems in these species.

Further, Table 1 also allows assessing the relationship between a given process and a given logistical structure. For example, cognitive biases are central to human models of pathological anxiety (e.g., [191]). Recent research has shown that the amygdala plays a central role in attentional biases towards threat in pathological anxiety [176]. As displayed in Table 1, it is interesting to note that empirical evidence has documented such cognitive biases only in species that have an amygdala. Hence, the present phylogenetic approach confirms that the amygdala plays a central role in cognitive biases observed in anxiety.

Different aspects of the literature reviewed above clearly suggest that anxiety as a conscious anticipation of danger only appears in great apes. This capacity, that implies autonoetic awareness, is directly related to the development of the neocortex and its connections with the limbic system and with the thalamus. This suggests that the capacity to represent oneself and one's reactions to hypothetical situations depends upon the capacity to strategically activate emotion networks or representations of emotional states. This reflexive capacity would be shared only by great apes and humans. Thus it might be that only great apes experience anxiety as humans, with its apprehension component. This does not mean that other species (e.g., other mammals such as rodents) may not have the aptitude to experiment anxiety with its anticipation dimension. However, in the case of lower mammals, this anticipation may not be conscious and may not be related to the ability to activate a representation of the situation with its possible consequences.

Finally, Table 1 also allows finding out the most relevant aspects of the anxiety response in a phylogenetic perspective. It thus seems that the coping appraisal check, the diversification of the emotional response, including the emotional expression and the physiological response, and the capacity for autonoetic awareness are the most relevant of these dimensions. Indeed, the coping potential ability enables us to separate insects from lower invertebrates, the diversification of the emotional response occurs at higher levels of the phylum (facial expressions appear in monkeys) and, finally, autonoetic consciousness appears in great apes.

To come back to our initial question, whether there is a qualitative difference between human and animal anxiety, Table 1 and our discussion of it suggest that it might not be the case. Rather, a clear phylogenetic trend appears, punctuated, thought, by important steps, as the three dimensions identified in the preceding paragraph. What is proper to human anxiety seems to be due to the well developed self-awareness capacity in that species. This feature, however, seems to be already shared, to a lesser extend, with great apes.

In conclusion, the present review proposes a general frame for discussing anxiety in the context of phylogeny. In many cases, the data necessary to assess the presence of a given process are not available and additional empirical work may be necessary to clarify this question. Still, as testified by the points highlighted in the general discussion, this approach proves to be heuristic, both for our understanding on how a phenomenon such as anxiety varies across the phylogeny, and for our understanding of the processes and logistic systems underlying anxiety.

## Figures and Tables

**Table 1:**
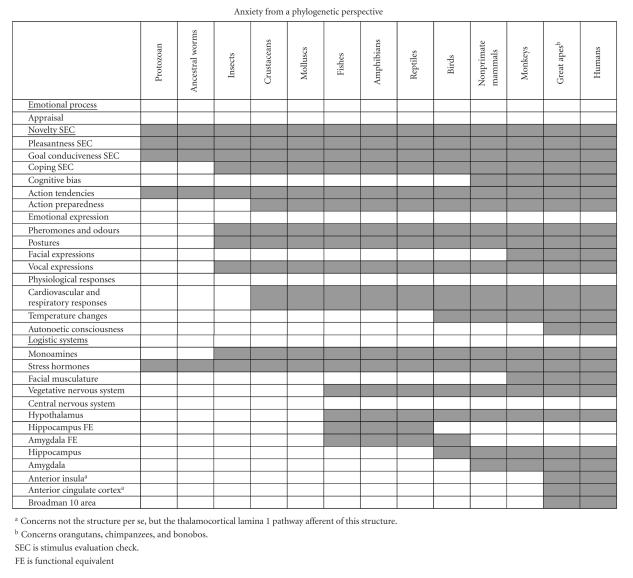
Summary of the findings about the presence of the different emotional responses and of the different logistic systems necessary for emotions across the phylum. Grey cells indicate presence of the process or system in a given phylum. SEC is stimulus evaluation check. FE is functional equivalent.

## References

[1] Barlow DH (2002). *Anxiety and Its Disorders: The Nature and Treatment of Anxiety and Panic*.

[2] Belzung C (2001). Rodent models of anxiety-like behaviors: are they predictive for compounds acting via non-benzodiazepine mechanisms?. *Current Opinion in Investigational Drugs*.

[3] Belzung C, Griebel G (2001). Measuring normal and pathological anxiety-like behaviour in mice: a review. *Behavioural Brain Research*.

[4] Belzung C, Chevalley C (2001). Models of complexity: the example of emotions. *Behavioral and Brain Sciences*.

[5] Ekman P, Scherer K, Ekman P (1984). Expression and the nature of emotion. *Approaches to Emotion*.

[6] Izard CE (1979). *Emotion in Personality and Psychopathology*.

[7] Greenberg LS (2002). *Emotion-Focused Therapy: Coaching Clients to Work through Their Feelings*.

[8] Damasio AR (1994). *Descartes' Error: Emotion, Reason and the Human Brain*.

[9] Blanchard RJ, Blanchard DC, Hori K, Blanchard RJ, Brain PF, Blanchard DC, Parmigiani S (1989). An ethoexperimental approach to the study of defense. *Ethoexperimental Approaches to the Study of Behaviour*.

[10] Frijda NH (1986). *The Emotions*.

[11] Scherer KR, Scherer K, Ekman P (1984). On the nature and function of emotion: a component process approach. *Approaches to Emotion*.

[12] Frijda NH, Kuipers P, ter Schure E (1989). Relations among emotion, appraisal, and emotional action readiness. *Journal of Personality and Social Psychology*.

[13] Darwin C (1965). *The Expression of Emotion in Man and Animals*.

[14] Tomkins SS, Plutchik R, Kellerman H (1980). Affect as amplification: some modification in theory. *Theories of Emotion*.

[15] von Uexküll J, Kriszat G (1934). *Streifzüge durch die Umwelten von Tieren und Menschen: Ein Bilderbuch unsichtbarer Welten*.

[16] Misslin R (2003). Une vie de cellule, forme et espace. *Revue de Synthèse*.

[17] Walters ET, Carew TJ, Kandel ER (1981). Associative learning in Aplysia: evidence for conditioned fear in an invertebrate. *Science*.

[18] Ruxton G (2006). Behavioural ecology: grasshoppers don't play possum. *Nature*.

[19] Schapker H, Breithaupt T, Shuranova Z, Burmistrov Y, Cooper RL (2002). Heart and ventilatory measures in crayfish during environmental disturbances and social interactions. *Comparative Biochemistry and Physiology—Part A: Molecular & Integrative Physiology*.

[20] Scherer KR, Dalgleish T, Power MJ (1999). Appraisal theory. *Handbook of Cognition and Emotion*.

[21] Wood DC (1969). Parametric studies of the response decrement produced by mechanical stimuli in the protozoan, *Stentor coeruleus*. *Journal of Neurobiology*.

[22] Rankin CH, Beck CDO, Chiba CM (1990). *Caenorhabditis elegans*: a new model system for the study of learning and memory. *Behavioural Brain Research*.

[23] Duerr JS, Quinn WG (1982). Three *Drosophila* mutations that block associative learning also affect habituation and sensitization. *Proceedings of the National Academy of Sciences of the United States of America*.

[24] Pinsker H, Kupfermann I, Castellucci V, Kandel E (1970). Habituation and dishabituation of the GM-withdrawal reflex in Aplysia. *Science*.

[25] Peeke HVS, Peeke SC, Peeke HVS, Herz MJ (1973). Habituation in fish with special reference to intraspecific aggressive behavior. *Habituation*.

[26] Davis M (1970). Effects of interstimulus interval length and variability on startle-response habituation in the rat. *Journal of Comparative and Physiological Psychology*.

[27] Geer JH (1966). Effect of interstimulus intervals and rest-period length upon habituation of the orienting response. *Journal of Experimental Psychology*.

[28] Schneirla T An evolutionary and developmental theory of biphasic processes underlying approach and withdrawal.

[29] Bargmann CI, Mori I, Riddle DL, Blumenthal T, Meyer BJ, Priess JR (1997). Chemotaxis and thermotaxis. *C. Elegans II*.

[30] Wittenburg N, Baumeister R (1999). Thermal avoidance in *Caenorhabditis elegans*: an approach to the study of nociception. *Proceedings of the National Academy of Sciences of the United States of America*.

[31] Zhang Y, Lu H, Bargmann CI (2005). Pathogenic bacteria induce aversive olfactory learning in *Caenorhabditis elegans*. *Nature*.

[32] Schwaerzel M, Monastirioti M, Scholz H, Friggi-Grelin F, Birman S, Heisenberg M (2003). Dopamine and octopamine differentiate between aversive and appetitive olfactory memories in *Drosophila*. *Journal of Neuroscience*.

[33] Dethier V, Stellar E (1970). *Animal Behavior*.

[34] Elliot AJ, Covington MV (2001). Approach and avoidance motivation. *Educational Psychology Review*.

[35] Schneirla T (1965). Aspects of stimulation and organization in approach/withdrawal processes underlying vertebrate behavioral development. *Advances in the Study of Behavior, Vol. 1*.

[36] de Bono M, Maricq AV (2005). Neuronal substrates of complex behaviors in *C. elegans*. *Annual Review of Neuroscience*.

[37] Hanlon RT, Messenger JB (1996). *Cephalopod Behaviour*.

[38] Honma A, Oku S, Nishida T (2006). Adaptive significance of death feigning posture as a specialized inducible defence against gape-limited predators. *Proceedings of the Royal Society B: Biological Sciences*.

[39] Miyatake T, Katayama K, Takeda Y, Nakashima A, Sugita A, Mizumoto M (2004). Is death-feigning adaptive? Heritable variation in fitness difference of death-feigning behaviour. *Proceedings of the Royal Society B: Biological Sciences*.

[40] Kusch J (1993). Behavioural and morphological changes in ciliates induced by the predator *Amoeba proteus*. *Oecologia*.

[41] Kusch J (1993). Induction of defensive morphological changes in ciliates. *Oecologia*.

[42] Kuhlmann HW, Kusch J, Heckmann K, Tollrian R, Harvell CD (1999). Predator-induced defenses in ciliated protozoa. *The Ecology and Evolution of Inducible Defenses*.

[43] Désiré L, Boissy A, Veissier I (2002). Emotions in farm animals: a new approach to animal welfare in applied ethology. *Behavioural Processes*.

[44] Désiré L, Veissier I, Després G, Boissy A (2004). On the way to assess emotions in animals: do lambs (Ovis aries) evaluate an event through its suddenness, novelty, or unpredictability?. *Journal of Comparative Psychology*.

[45] Overmier JB, Seligman ME (1967). Effects of inescapable shock upon subsequent escape and avoidance responding. *Journal of Comparative and Physiological Psychology*.

[46] Braud W, Wepman B, Russo D (1969). Task and species generality of the ‘helplessness’ phenomenon. *Psychonomic Science*.

[47] Hiroto D (1974). Locus of control and learned helplessness. *Journal of Experimental Psychology*.

[48] Maier SF, Seligman MEP, Solomon RL, Campbell BA, Church RM (1969). Pavlovian fear conditioning and learned helplessness: effects on escape and avoidance behavior of: (a) the CS-US contingency; and (b) the independence of the US and voluntary responding. *Punishment and Aversive Behavior*.

[49] Overmier JB (1968). Interference with avoidance behavior: failure to avoid traumatic shock. *Journal of Experimental Psychology*.

[50] Seligman MEP, Beagley G (1975). Learned helplessness in the rat. *Journal of Comparative and Physiological Psychology*.

[51] Seligman MEP, Maier SF, Geer JH (1968). Alleviation of learned helplessness in the dog. *Journal of Abnormal Psychology*.

[52] Seligman MEP, Maier SF, Solomon RL, Brush FR (1971). Unpredictable and uncontrollable aversive events. *Aversive Conditioning and Learning*.

[53] Seligman MEP, Rosellini RA, Kozak MJ (1975). Learned helplessness in the rat: time course, immunization, and reversibility. *Journal of Comparative and Physiological Psychology*.

[54] Behrend ER, Bitterman ME (1963). Sidman avoidance in the fish. *Journal of the Experimental Analysis of Behavior*.

[55] Pinckney GA (1967). Avoidance learning in fish as a function of prior fear conditioning. *Psychological Reports*.

[56] Padilla AM, Padilla C, Ketterer T, Giaealone D (1970). Inescable shocks and subsequent escape/avoidance conditioning in goldfish, Carassius auratus. *Psychonomic Science*.

[57] Brown GE, Stroup K (1988). Learned helplessness in the cockroach (*Periplaneta americana*). *Behavioral and Neural Biology*.

[58] Brown GE, Busby PL, Klopfenstein MK (1992). Decay and reversibility of learned helplessness in the cockroach (*Periplaneta americana*). *Psychological Reports*.

[59] Brown GE, Davenport DA, Howe AR (1994). Escape deficits induced by a biologically relevant stressor in the slug (Limax maximus). *Psychological Reports*.

[60] Perry S, Manson JH (2003). Traditions in monkeys. *Evolutionary Anthropology*.

[61] Imanishi K, Imanishi K (1952). The evolution of human nature. *Man*.

[62] Huffman MA, Heyes CM, Galef BG (1996). Acquisition of innovative cultural behaviors in nonhuman primates: a case study of stone handling, a socially transmitted behaviour in Japanese macaques. *Social Learning in Animals*.

[63] Whiten A, Goodall J, McGrew WC (1999). Cultures in chimpanzees. *Nature*.

[64] Donald M (1993). Précis of origins of the modern mind: three stages in the evolution of culture and cognition. *Behavioral and Brain Sciences*.

[65] Blackmore S (1999). *The Meme Machine*.

[66] Tomasello M, Kruger AC, Ratner HH (1993). Cultural learning. *Behavioral and Brain Sciences*.

[67] Stemmler G, Philippot P, Feldman RS (2004). Physiological processes during emotion. *The Regulation of Emotion*.

[68] Pauls CA, Philippot P, Feldman RS (2004). Physiological consequences of emotion regulation: taking into accouth the effects of strategies, personality and situation. *The Regulation of Emotion*.

[69] Bandler R, Keay KA, Floyd N, Price J (2000). Central circuits mediating patterned autonomic activity during active vs. passive emotional coping. *Brain Research Bulletin*.

[70] Lang PJ, Davis M, Öhman A (2000). Fear and anxiety: animal models and human cognitive psychophysiology. *Journal of Affective Disorders*.

[71] Maren S (2001). Nuerobiology of Pavlovian fear conditioning. *Annual Review of Neuroscience*.

[72] Carrasco GA, van de Kar LD (2003). Neuroendocrine pharmacology of stress. *European Journal of Pharmacology*.

[73] Johnsson JI, Höjesjö J, Fleming IA (2001). Behavioural and heart rate responses to predation risk in wild and domesticated Atlantic salmon. *Canadian Journal of Fisheries and Aquatic Sciences*.

[74] Baker MA, Cronin MJ, Mountjoy DG (1976). Variability of skin temperature in the waking monkey. *American Journal of Physiology*.

[75] Wells MJ (1978). *Octopus. Physiology and Behaviour of an Advanced Invertebrate*.

[76] Astley CA, Smith OA, Ray RD (1991). Integrating behavior and cardiovascular responses: the code. *American Journal of Physiology*.

[77] Elfenbein HA, Hess U, Philippot P (2006). It takes one to know one better: controversy about cultural in-group advantage in communicating emotion as a theoretical rather than methodological issue. *Group Dynamics and Emotional Expression*.

[78] Matsumoto D (1987). The role of facial response in the experience of emotion: more methodological problems and a meta-analysis. *Journal of Personality and Social Psychology*.

[79] Izard CE (1990). Facial expressions and the regulation of emotions. *Journal of Personality and Social Psychology*.

[80] Bachorowski J-A (1999). Vocal expression and perception of emotion. *Current Directions in Psychological Science*.

[81] Bachorowski J-A, Owren MJ (2003). Sounds of emotion: production and perception of affect-related vocal acoustics. *Annals of the New York Academy of Sciences*.

[82] Scherer KR, Kappas A, Todt D, Goedeking P, Symmes D (1988). Primate vocal expression of affective state. *Primate Vocal Communication*.

[83] Hatfield E, Cacioppo JT, Rapson RL (1993). *Emotional Contagion*.

[84] Hess U, Philippot P, Blairy S (1998). Facial reactions to emotional facial expressions: affect or cognition?. *Cognition and Emotion*.

[85] Sullins ES (1991). Emotional contagion revisited: effects of social comparison and expressive style on mood convergence. *Personality and Social Psychology Bulletin*.

[86] Duclos SD, Laird JD, Schneider E, Sexter M, Stern L, VanLighten O (1989). Emotion-specific effects of facial expressions and postures on emotional experience. *Journal of Personoliry and Social Psychology*.

[87] Stepper S, Strack F (1993). Proprioceptive determinants of affective and nonaffective feelings. *Journal of Personality and Social Psychology*.

[88] Chen D, Haviland-Jones J (2000). Human olfactory communication of emotion. *Perceptual and Motor Skills*.

[89] Huber E (1931). *Evolution of Facial Musculature and Facial Expression*.

[90] Burrows AM, Waller BM, Parr LA, Bonar CJ (2006). Muscles of facial expression in the chimpanzee (*Pan troglodytes*): descriptive, comparative and phylogenetic contexts. *Journal of Anatomy*.

[91] Berridge KC (2000). Measuring hedonic impact in animals and infants: microstructure of affective taste reactivity patterns. *Neuroscience and Biobehavioral Reviews*.

[92] Andrew RJ (1963). Evolution of facial expression. *Science*.

[93] Andrew RJ (1963). The origin and evolution of the calls and facial expressions of the primates. *Behaviour*.

[94] Seyfarth RM, Cheney DL, Marler P (1980). Monkey responses to three different alarm calls: evidence of predator classification and semantic communication. *Science*.

[95] Randall JA, McCowan B, Collins KC, Hooper SL, Rogovin K (2005). Alarm signals of the great gerbil: acoustic variation by predator context, sex, age, individual, and family group. *Journal of the Acoustical Society of America*.

[96] Gyger M, Karakashian SJ, Dufty AM, Marler P (1988). Alarm signals in birds: the role of testosterone. *Hormones and Behavior*.

[97] Staton MA (1978). “Distress calls” of crocodilians—whom do they benefit?. *The American Naturalists*.

[98] Smith RJF (1992). Alarm signals in fishes. *Reviews in Fish Biology and Fisheries*.

[99] Wyttenbach RA, May ML, Hoy RR (1996). Categorical perception of sound frequency by crickets. *Science*.

[100] Maschwitz UW (1966). Alarm substances and alarm behavior in social insects. *Vitamins and Hormones*.

[101] Pruitt CH, Burghardt GM, Sebeok TA (1977). Communication in terrestrial carnivores: Mustelidae, Procyonidae, and Ursidae. *How Animals Communicate*.

[102] Valenta JG, Rigby MK, Doty RL (1976). Discrimination of the odor of stressed rats. *Mammalian Olfaction, Reproductive Processes, and Behavior*.

[103] Manley GH, Doty RL (1976). Functions of the external genital glands of Perodicticus and Arctocebus. *Mammalian Olfaction, Reproductive Processes, and Behavior*.

[104] Cott HB (1940). *Adaptive Coloration in Animals*.

[105] Edmunds M (1974). *Defence in Animals*.

[106] Endler JA (1978). A predator's view of animal color patterns. *Evolutionary Biology*.

[107] Dice L, Blossom PM (1937). Studies of mammalian ecology in southwestern North America, with special attention to the colors of desert mammals. *Publications of the Carnegie Institution of Washington*.

[108] Santos JC, Coloma LA, Cannatella DC (2003). Multiple, recurring origins of aposematism and diet specialization in poison frogs. *Proceedings of the National Academy of Sciences of the United States of America*.

[109] Yiend J (2004). *Cognition, Emotion and Psychopathology: Theoretical, Empirical and Clinical Directions*.

[110] Mogg K, Bradley BP, Yiend J (2004). A cognitive-motivational perspective on the processing of threat information and anxiety. *Cognition, Emotion and Psychopathology: Theoretical, Empirical and Clinical Directions*.

[111] Winslow JT, Parr LA, Davis M (2002). Acoustic startle, prepulse inhibition, and fear-potentiated startle measured in rhesus monkeys. *Biological Psychiatry*.

[112] Davis M (1986). Pharmacological and anatomical analysis of fear conditioning using the fear-potentiated startle paradigm. *Behavioral Neuroscience*.

[113] Falls WA, Carlson S, Turner JG, Willott JF (1997). Fear-potentiated startle in two strains of inbred mice. *Behavioral Neuroscience*.

[114] Beuzen A, Belzung C (1995). Link between emotional memory and anxiety states: a study by principal component analysis. *Physiology and Behavior*.

[115] Venault P, Chapouthier G, de Carvalho LP (1986). Benzodiazepine impairs and *β*-carboline enhances performance in learning and memory tasks. *Nature*.

[116] Belzung C, Le Guisquet AM, Griebel G (2000). *β*-CCT, a selective BZ-*ω*1 receptor antagonist, blocks the anti-anxiety but not the amnesic action of chlordiazepoxide in mice. *Behavioural Pharmacology*.

[117] Ducottet C, Aubert A, Belzung C (2004). Susceptibility to subchronic unpredictable stress is related to individual reactivity to threat stimuli in mice. *Behavioural Brain Research*.

[118] Crestani F, Lorez M, Baer K (1999). Decreased GABA_A_-receptor clustering results in enhanced anxiety and a bias for threat cues. *Nature Neuroscience*.

[119] Laird JD (1989). Mood affects memory because feelings are cognitions. *Journal of Social Behavior and Personality*.

[120] Lane RD, Lane RD, Nadel L (2000). Neural correlates of conscious emotional experience. *Cognitive Neuroscience of Emotion*.

[121] Wheeler MA, Stuss DT, Tulving E (1997). Toward a theory of episodic memory: the frontal lobes and autonoetic consciousness. *Psychological Bulletin*.

[122] Tulving E (1984). Précis of elements of episodic memory. *The Behavioral and Brain Sciences*.

[123] Roberts WA (2002). Are animals stuck in time?. *Psychological Bulletin*.

[124] Zentall TR, Clement TS, Bhatt RS, Allen J (2001). Episodic-like memory in pigeons. *Psychonomic Bulletin and Review*.

[125] Gallup GG, Anderson JR, Platek SM, Kircher T, David A (2003). Self-awareness, social intelligence and schizophrenia. *The Self in Neuroscience and Psychiatry*.

[126] Gallop GG (1970). Chimpanzees: self-recognition. *Science*.

[127] Povinelli DJ (1987). Monkeys, apes, mirrors and minds: the evolution of self-awareness in primates. *Human Evolution*.

[128] Suarez SD, Gallup GG (1981). Self-recognition in chimpanzees and orangutans, but not gorillas. *Journal of Human Evolution*.

[129] Westergaard GC, Hyatt CW (1994). The responses of bonobos (*Pan paniscus*) to their mirror images: evidence of self-recognition. *Human Evolution*.

[130] Ledbetter DH, Basen JA (1982). Failure to demonstrate self-recognition in gorillas. *American Journal of Primatology*.

[131] Anderson JR (1999). Primates and representations of self. *Current Psychology of Cognition*.

[132] Heyes CM (1998). Theory of mind in nonhuman primates. *Behavioral and Brain Sciences*.

[133] Hare B, Call J, Agnetta B, Tomasello M (2000). Chimpanzees know what conspecifics do and do not see. *Animal Behaviour*.

[134] Norris DO (1985). *Vertebrate Endocrinology*.

[135] Robertson HA, Juorio AV (1976). Octopamine and some related noncatecholic amines in invertebrate nervous systems. *International Review of Neurobiology*.

[136] Goodrich JT, Bernd P, Sherman D, Gershon MD (1980). Phylogeny of enteric serotonergic neurons. *Journal of Comparative Neurology*.

[137] Peroutka SJ (1992). Phylogenetic tree analysis of G protein-coupled 5-HT receptors: implications for receptor nomenclature. *Neuropharmacology*.

[138] Hen R (1993). Structural and functional conservation of serotonin receptors throughout evolution. *EXS*.

[139] Csaba G (1993). Presence in and effects of pineal indoleamines at very low level of phylogeny. *Experientia*.

[140] Weiger WA (1997). Serotonergic modulation of behaviour: a phylogenetic overview. *Biological Reviews of the Cambridge Philosophical Society*.

[141] Lacoste A, Malham SK, Cueff A, Poulet SA (2001). Stress-induced catecholamine changes in the hemolymph of the oyster Crassostrea gigas. *General and Comparative Endocrinology*.

[142] Ottaviani E, Franceschi C (1996). The neuroimmunology of stress from invertebrates to man. *Progress in Neurobiology*.

[143] Nilsson S (1983). *Autonomic Nerve Function in the Vertebrates*.

[144] Nilsson S, Holmgren S, Burnstock G (1994). Comparative physiology and evolution of the autonomic nervous system. *The Autonomic Nervous System*.

[145] Zavarzin AA (1941). *Ocherki po evol'utsionnoj gistologii nervnoj sistemy [Essays on the Evolutionary Histology of the Nervous System]*.

[146] Sherwood CC, Hof PR, Holloway RL (2005). Evolution of the brainstem orofacial motor system in primates: a comparative study of trigeminal, facial, and hypoglossal nuclei. *Journal of Human Evolution*.

[147] Sherwood CC (2005). Comparative anatomy of the facial motor nucleus in mammals, with an analysis of neuron numbers in primates. *The Anatomical Record—Part A: Discoveries in Molecular, Cellular, and Evolutionary Biology*.

[148] Huber E (1930). Evolution of facial musculature and cutaneous field of trigeminus—part I. *Quarterly Review of Biology*.

[149] LaBar KS, Gatenby JC, Gore JC, LeDoux JE, Phelps EA (1998). Human amygdala activation during conditioned fear acquisition and extinction: a mixed-trial fMRI study. *Neuron*.

[150] Calder AJ, Lawrence AD, Young AW (2001). Neuropsychology of fear and loathing. *Nature Reviews Neuroscience*.

[151] Knight DC, Cheng DT, Smith CN, Stein EA, Helmstetter FJ (2004). Neural substrates mediating human delay and trace fear conditioning. *The Journal of Neuroscience*.

[152] Gray M, Kemp AH, Silberstein RB, Nathan PJ (2003). Cortical neurophysiology of anticipatory anxiety: an investigation utilizing steady state probe topography (SSPT). *NeuroImage*.

[153] Chua P, Krams M, Toni I, Passingham R, Dolan R (1999). A functional anatomy of anticipatory anxiety. *NeuroImage*.

[154] Reiman EM, Fusselman MJ, Fox PT, Raichle ME (1989). Neuroanatomical correlates of anticipatory anxiety. *Science*.

[155] Damasio AR, Grabowski TJ, Bechara A (2000). Subcortical and cortical brain activity during the feeling of self-generated emotions. *Nature Neuroscience*.

[156] Northcutt RG, Braford MR, Ebbesson SOE (1980). New observations on the organization and evolution of the telencephalon of actinopterygian fishes. *Comparative Neurology of the Telencephalon*.

[157] Nieuwenhuys R, Meek J, Jones EG, Peters A (1990). The telencephalon of actinopterygian fishes. *Comparative Structure and Evolution of the Cerebral Cortex*.

[158] Braford MR (1995). Comparative aspects of forebrain organization in the ray-finned fishes: touchstones or not?. *Brain, Behavior and Evolution*.

[159] Butler AB (2000). Topography and topology of the teleost telencephalon: a paradox resolved. *Neuroscience Letters*.

[160] Portavella M, Vargas JP (2005). Emotional and spatial learning in goldfish is dependent on different telencephalic pallial systems. *European Journal of Neuroscience*.

[161] Portavella M, Vargas JP, Torres B, Salas C (2002). The effects of telencephalic pallial lesions on spatial, temporal, and emotional learning in goldfish. *Brain Research Bulletin*.

[162] Portavella M, Torres B, Salas C (2004). Avoidance response in goldfish: emotional and temporal involvement of medial and lateral telencephalic pallium. *The Journal of Neuroscience*.

[163] Bruce LL, Neary TJ (1995). The limbic system of tetrapods: a comparative analysis of cortical and amygdalar populations. *Brain, Behavior and Evolution*.

[164] Moreno N, González A (2006). The common organization of the amygdaloid complex in tetrapods: new concepts based on developmental, hodological and neurochemical data in anuran amphibians. *Progress in Neurobiology*.

[165] Moreno N, Morona R, López JM, Muñoz M, González A (2005). Lateral and medial amygdala of anuran amphibians and their relation to olfactory and vomeronasal information. *Brain Research Bulletin*.

[166] Laberge F, Mühlenbrock-Lenter S, Grunwald W, Roth G (2006). Evolution of the amygdala: new insights from studies in amphibians. *Brain, Behavior and Evolution*.

[167] Lanuza E, Font C, Martínez-Marcos A, Martínez-García F (1997). Amygdalo-hypothalamic projections in the lizard Podarcis hispanica: a combined anterograde and retrograde tracing study. *Journal of Comparative Neurology*.

[168] Novejarque A, Lanuza E, Martínez-García F (2004). Amygdalostriatal projections in reptiles: a tract-tracing study in the lizard Podarcis hispanica. *Journal of Comparative Neurology*.

[169] Székely AD (1999). The avian hippocampal formation: subdivisions and connectivity. *Behavioural Brain Research*.

[170] Cheng M-F, Chaiken M, Zuo M, Miller H (1999). Nucleus taenia of the amygdala of birds: anatomical and functional studies in ring doves (*Streptopelia risoria*) and European starlings (*Sturnus vulgaris*). *Brain, Behavior and Evolution*.

[171] Maren S (2003). The amygdala, synaptic plasticity, and fear memory. *Annals of the New York Academy of Sciences*.

[172] Maren S, Holt W (2000). The hippocampus and contextual memory retrieval in Pavlovian conditioning. *Behavioural Brain Research*.

[173] Gewirtz JC, McNish KA, Davis M (2000). Is the hippocampus necessary for contextual fear conditioning?. *Behavioural Brain Research*.

[174] Anagnostaras SG, Gale GD, Fanselow MS (2001). Hippocampus and contextual fear conditioning: recent controversies and advances. *Hippocampus*.

[175] LeDoux JE (2000). Emotion circuits in the brain. *Annual Review of Neuroscience*.

[176] LeDoux JE (2003). The emotional brain, fear, and the amygdala. *Cellular and Molecular Neurobiology*.

[177] Phelps EA, LeDoux JE (2005). Contributions of the amygdala to emotion processing: from animal models to human behavior. *Neuron*.

[178] Schafe GE, Nader K, Blair HT, LeDoux JE (2001). Memory consolidation of Pavlovian fear conditioning: a cellular and molecular perspective. *Trends in Neurosciences*.

[179] Dielenberg RA, McGregor IS (2001). Defensive behavior in rats towards predatory odors: a review. *Neuroscience and Biobehavioral Reviews*.

[180] Blanchard DC, Canteras NS, Markham CM, Pentkowski NS, Blanchard RJ (2005). Lesions of structures showing FOS expression to cat presentation: effects on responsivity to a Cat, Cat odor, and nonpredator threat. *Neuroscience and Biobehavioral Reviews*.

[181] Nieuwenhuys R, ten Donkelaar HJ, Nicholson C (1998). *The Central Nervous System of Vertebrates*.

[182] Northcutt RG, Kaas JH (1995). The emergence and evolution of mammalian neocortex. *Trends in Neurosciences*.

[183] Karten HJ (1997). Evolutionary developmental biology meets the brain: the origins of mammalian cortex. *Proceedings of the National Academy of Sciences of the United States of America*.

[184] Heidbreder CA, Groenewegen HJ (2003). The medial prefrontal cortex in the rat: evidence for a dorso-ventral distinction based upon functional and anatomical characteristics. *Neuroscience and Biobehavioral Reviews*.

[185] Singewald N, Salchner P, Sharp T (2003). Induction of c-Fos expression in specific areas of the fear circuitry in rat forebrain by anxiogenic drugs. *Biological Psychiatry*.

[186] Critchley HD, Wiens S, Rotshtein P, Öhman A, Dolan RJ (2004). Neural systems supporting interoceptive awareness. *Nature Neuroscience*.

[187] Critchley HD (2004). The human cortex responds to an interoceptive challenge. *Proceedings of the National Academy of Sciences of the United States of America*.

[188] Craig AD (2002). How do you feel? Interoception: the sense of the physiological condition of the body. *Nature Reviews Neuroscience*.

[189] Nimchinsky EA, Gilissen E, Allman JM, Perl DP, Erwin JM, Hof PR (1999). A neuronal morphologic type unique to humans and great apes. *Proceedings of the National Academy of Sciences of the United States of America*.

[190] Allman J, Hakeem A, Watson K (2002). Two phylogenetic specializations in the human brain. *Neuroscientist*.

[191] Clark DM, Wells A, Heimberg R, Liebowitz M, Hope DA, Schneier FR (1995). A cognitive model of social phobia. *Social Phobia: Diagnosis, Assessment and Treatment*.

